# An emerging role of inflammatory biomarker (suPAR) for prognostic evaluation of atrial fibrillation

**DOI:** 10.1002/joa3.70116

**Published:** 2025-06-16

**Authors:** Nihar Jena

**Affiliations:** ^1^ Department of Interventional Cardiology Marshall University Huntington West Virginia USA

**Keywords:** atrial fibrillation, suPAR

To the Editor,

Atrial fibrillation (AFib) is a chronic cardiovascular condition that poses a significant global challenge due to difficulties in identification and treatment. The global burden of AFib and atrial flutter has been rising over the past decade, with a prevalence of 52.5 million reported in 2021 and 10.55 million reported in the United States in 2019.^1^ Although various risk scores, such as CHAD2‐VASc and HAS‐BLED, as well as inflammatory biomarkers like C‐reactive protein (CRP), are available, there is a need for further research in this area. This research could enhance our ability to prognosticate both short‐term and long‐term outcomes for individuals with AFib. Wisborg et al.^2^ provided a timely and thought‐provoking prospective analysis of AFib patients admitted to the emergency department with elevated soluble urokinase plasminogen activator receptor (suPAR) levels, showing an increased all‐cause mortality at 1 year.

The novel biomarker suPAR has been identified as a stable biomarker for cardiovascular disease associated with chronic inflammation. suPAR is a cleaved product of plasminogen activator receptor and a component of the fibrinolytic system, which is usually formed due to an immunological response to inflammation from the cell surface.^3^ Various studies have shown promising results for predicting long‐term cardiovascular outcomes like acute myocardial infarction, atherosclerosis, and coronary calcifications.^4^, ^5^ In a study by Ichihara, the prevalence of suPAR level was shown to be higher in nonparoxysmal AFib patients.^6^ This prospective cohort study, comprising 339 patients, showed higher mortality in the patient group with a higher level of suPAR. The authors depicted a 12% mortality increase per unit ng/mL increase in suPAR despite adjusting for age, sex, smoking, creatinine, and another inflammatory biomarker like CRP.

This study highlights the importance of suPAR in AFib for several compelling reasons. First, stratifying high‐risk AFib patients based on a higher level of suPAR can help determine the level of care and predict outcomes in patients presenting to the emergency department or inpatient setting. Second, suPAR has a longer half‐life than other cardiac biomarkers, making it a more reliable long‐term prognostic marker, especially given the often delayed presentation and chronic nature of AFib.

The study is well‐designed and has an adequate sample size with a statistically significant outcome. The statistical modeling is robust, and the variables were adjusted using multivariate Cox regression, along with adjustments for other variables. Nonetheless, the major limitation is that the study is a prospective analysis in a single center, so generalizability is questionable. Furthermore, the cause of death was not pinpointed to cardiovascular etiology, making it challenging to determine whether the patient died from any other reason. Additionally, suPAR is a nonspecific biomarker, so its validity in patients with AFib and concurrent inflammatory diseases will be limited.

It is commendable that Wisborg et al. have reignited the discussion surrounding biomarkers for assessing long‐term prognosis in AFib. A growing focus has been placed lately on the identification and treatment of AFib. Studies like this definitely illuminate the risk stratification and timely management of patients to change their outcomes. The incorporation of biomarkers in AFib management could be practice changing if more robust data from randomized trials become available. Looking ahead, future studies can determine whether targeted anti‐inflammatory agents can be incorporated into individualized patient‐tailored strategies for AFib management.

## CONFLICT OF INTEREST STATEMENT

None.
